# The commoditization of civil nuclear power

**DOI:** 10.1098/rsos.240021

**Published:** 2024-05-22

**Authors:** Eann A. Patterson, Richard J. Taylor

**Affiliations:** ^1^ Department of Mechanical and Aerospace Engineering, University of Liverpool, Liverpool, UK; ^2^ Dalton Nuclear Institute, University of Manchester, Manchester, UK

**Keywords:** micro-power unit, micro-modular reactors, MMRs, nuclear power, integrated nuclear digital environment, INDE

## Abstract

The commoditization of nuclear power through the factory production of sealed micro-power units within a digitally enabled holistic assurance framework is described. This would revolutionize nuclear power-plant design, construction, operation and decommissioning through a paradigm shift to manufacture–operate–remove–recycle (MORR). The potential impact of recent research on an integrated nuclear digital environment for large bespoke nuclear power plants and the design, build and operation of fusion power plants using such a digital environment is explored. These strands are interwoven to discuss the technical, economic and socio-political implications of MORR in the context of micro-reactors and to consider the potential evolution of safeguarding issues based on a digital assurance framework that leads to type approvals. Commoditization of nuclear power would lower costs in line with offshore wind and the output from a single production line in a factory could replace a third of current fossil fuel-based electricity generation in the UK over a 15-year period, making a significant contribution to achieving zero greenhouse gas emissions. The challenges associated with the changes in culture, both in the nuclear industry and in society, as well as the technology gaps, that need to be addressed in realizing this paradigm shift are identified and discussed.

## 1. Introduction

June 2023 was the first June on record when the daily average global 2-m surface temperature exceeded 1.5°C above pre-industrial levels, suggesting global temperatures are rising faster than anticipated [1]. In 2020, it was estimated that the world’s remaining carbon budget was about 500 Gt CO_2_ if there was to be a 50% probability of limiting global warming to 1.5°C above pre-industrial levels. Three years later, the remaining budget was estimated to be about 250 Gt CO_2_, i.e. it halved in 3 years, as a result of continued pollution and temperatures rising faster than anticipated [2]. Therefore, there is an imperative to massively accelerate progress towards net zero emissions and to start measures to ameliorate the impact of rising global temperatures. This need has been translated into targets for zero carbon emissions enshrined in the Paris Accord and Glasgow Climate Pact, which are ambitious for most industrialized nations in terms of energy production and consumption because of the need to find substitutes for fossil fuel as the prime energy source for space heating and transport, at the same time as replacing fossil-fuelled and ageing power stations. Electricity and hydrogen offer potential routes to distribute energy but both require sources of energy generation that have close-to-zero carbon footprints and nuclear power could be a serious contender if its costs and timescales were competitive.

Globally, the main economic sectors contributing to global greenhouse gas emissions are electricity and heat production (25%); agriculture, forestry and other land use (24%); industry (21%); transportation (14%); other energy (10%) and buildings (6%) [3]. In Japan, the UK and the USA, transportation using fossil fuels accounts for 32%, 31% and 25%, respectively, of national energy consumption while electricity output accounts for 38%, 21% and 13% [4–6]. If all vehicles were powered by electricity, either directly or indirectly via hydrogen, then this implies power station output would need to increase between twofold and threefold in these countries. A similar analysis can be performed for space heating, which in many countries is mainly powered by fossil fuels. Hence, there is an urgent need not only to replace fossil-fuel power stations that are responsible for about a quarter of global greenhouse gas emissions but also to increase electricity-generating capacity globally. It is impractical for any single green (i.e. net zero emissions) source of energy to replace fossil fuels, and hence a blend of sources will be needed. Nuclear energy could play a significant role if the timescales and costs for manufacturing nuclear power units were similar to those for other green sources of energy, such as wind.

The median construction time for nuclear reactors completed in 2019 was just under 10 years [7], which typically consists of 5 years for planning and approvals prior to construction assuming there is no political or societal opposition and 5 years for construction and commissioning [8,9] assuming that there are no construction delays. Construction delays are not unusual with three-quarters of new light water reactors being delayed during construction. If the plant was completed overnight so that no interest was incurred, then overnight construction costs (OCCs) would be between 3000 and 4000 US$_2023_  kWe^−1^ [10]. This is comparable to the capital expenditure for offshore wind turbines in California [11] but considerably higher than for land-based wind turbines which is about 1500 US$_2023_  MWe^−1^ [12]. The unit size of nuclear reactors, typically 700 MWe, and the long construction period increase the price of the capital required [13]. Hence, there is a drive towards smaller power units and faster construction, which has led to small modular reactors (SMRs) being proposed. The output of SMRs is typically less than 300 MWe, and they are manufactured in a factory for assembly either on-site or in the factory for transportation to the site. Typically, micro-modular reactors (MMRs), generally regarded as a sub-set of SMRs with outputs up to about 20 MWe, are designed for factory production and transport to the site where power is required, though no factories exist at the moment.

A key advantage of MMRs is the option to design for hazards with only on-site, rather than off-site, consequences, i.e. a US DoE classification of Hazard Category 2 [14]. This implies complete containment of any incident in the reactor, i.e. an ‘in-the-box’ approach. This approach is already being explored for fusion power plants and is viable for small-scale fission plants with micro-reactors up to 20 MWe. Several countries have developed prototype fission reactors of this size. For example, Mitsubishi is planning prototype tests between 2026 and 2030 on micro-reactors with an ‘in-the-box’ or ‘in-the-container’ approach to safety [15]; while Westinghouse is designing their eVinci transportable, mobile micro-reactor [16]. Hence, the challenge lies not in the technical feasibility of the design, but in designing for factory production and providing a robust quality management system that generates trust and confidence among the supply chain, regulatory authorities and the general public. These challenges are discussed in this article.

## 2. Current status

The focus of most previous research on nuclear reactors for power generation has been on reactor physics, materials science and safety considerations of existing design concepts that are decades old. Design for manufacture at a competitive cost has been a secondary consideration with a risk-averse safety culture often inhibiting innovative thinking and developments. Recent advances in digital engineering, including the integrated nuclear digital environment (INDE) [17] and its application to the design of a fusion energy plant [18], provide an opportunity to explore new design spaces and investigate game-changing approaches to the design, manufacture, safety and use of nuclear reactors.

There is a substantial body of research on the design of SMRs, which has been reviewed recently including by Hussein [19] in 2020, the OECD [20] in 2021 and in 2023 by Tan *et al.* [21] and by Fernández-Arias *et al.* [22]. There is also a developing scientific literature on manufacturing systems and technology for nuclear power stations; for example, an editorial on ‘Advanced manufacturing for nuclear energy’ concluded that new ways to manufacture nuclear components were being sought to reduce costs and development times [23]. However, these studies relate to ‘job’ production rather than ‘flow’ production, i.e. the construction of single nuclear power plants, usually tailored to their geographical location, as opposed to factory or flow production. Modular designs for reactors provide the opportunity to reduce construction costs and achieve greater uniformity between sites; however, at the moment, they are deployed as a single ‘job’ with regulatory approval required for each site, which tends to offset reductions in development times and costs. MMRs are at the design stage in the UK (U-Battery (4 MWe) by Urenco and partners) and in the USA (eVinci (0.2–5 MWe) by Westinghouse), while a licence application has been submitted to US NRC by Oklo for their Aurora reactor (2 MWe). The Ultra Safe Nuclear Corporation (USNC) has proposed demonstration micro-reactors (5–10 MWe) at the University of Illinois Urbana-Champaign and Chalk River, Ontario to be constructed by 2026 [24] and Mitsubishi plans prototype tests on its micro-reactor, with an ‘in-the-box’ safety philosophy, on the same timescale [25].

Lovering *et al.* [26] have shown that micro-reactors could be competitive with diesel generators provided capital costs are controlled by building hundreds of identical units which would be enabled by factory-based, flow production. However, almost no research has been published on factory-based production, the use of digital environments or threads and blockchains, or supply chain and business models for factory-based production of nuclear power plants. Digital threads (links between real-world systems and their cyber representations) are becoming an important component of manufacturing processes as described recently by Hedburg *et al.* [27,28], Kwon *et al.* [29] and Kurfess & Grimes [30]. Hence, it is worthwhile to consider the benefits these technologies could offer to the civil nuclear power industry.

## 3. Future blueprint

Our hypothesis is that these technologies can deliver the commoditization of nuclear power via a factory-based regulatory regime based on a digitally enabled holistic assurance framework that will enable the global deployment of micro power units with costs and timescales comparable to those of other green sources of electricity. For example, it should be feasible to produce micro power units at approximately the same rate of production as a large passenger aircraft, e.g. one per week for an Airbus A350. This implies that four such production lines, assembling a 20 MWe unit per week, would produce sufficient generating capacity to replace the current fossil fuel-based UK electricity generating capacity (44.4 GWe [31]) in slightly less than 12 years, i.e. the median construction time for a single large nuclear reactor. This size of micro-reactor (approximately 20 MWe ≅ 60 000 households), and its associated plant, could have a small physical footprint equating to a collection of shipping containers and be classified as Hazard Category 2 in the US DoE classification, i.e. hazards with on-site rather than off-site consequences through the use of an ‘in-the-box’ safety philosophy, thus allowing type approval by regulators following the example of the aircraft industry. A sealed and secure micro-power unit, which is returned to the factory for maintenance, refuelling and recycling, would remove nuclear-safeguarding issues from the generating site to the factory and allow this completely different regulatory approach, based on approving the design, production and quality assurance processes for a type or series. Trust would need to be established with the supply chain, where components will need to be delivered to the appropriate, and as yet undefined standards, through to the end-user and general public. The relationship with the public would also need to change, to allow acceptance of clusters of containers (more akin to Airstream caravans, than e.g. shipping containers) in their neighbourhood, so that such units would be perceived as a commonplace, climate-friendly commodity, disassociated from its fundamental technology.

These factory-built nuclear power units, installed singly or clustered in microgrids, would be a century-defining product that could be deployed in urban environments, in remote locations or at industrial sites to provide electricity or to generate hydrogen without creating nuclear-licensed sites; and, given their physical size, could be readily transported back to the factory for maintenance, refuelling and recycling. This production rate, maintenance schedule and safety regime represent a paradigm change for the nuclear industry that requires a change in design philosophy, development of a digitally assured supply chain and production system, and will lead to the commoditization of nuclear energy.

## 4. Economic, socio-political and technical implications

### 4.1. Economic implications

Our blueprint represents a paradigm shift for the nuclear industry in terms of the design, production and operation of nuclear power plants that could reverse both its unpromising economic prospects [32] and its poor reputation in society [33,34]. The latter two are intertwined because taxpayers’ money is often used to support economically unviable construction and decommissioning of nuclear power plants and because public opinion, swayed by association with legacy nuclear waste and nuclear weapons, often does not support these processes. Hence, it is crucial from the outset that a financially viable business model is identified and explored at a high level, considering the manufacturing, deployment, maintenance/refuelling and decommissioning of micro-power units. The blueprint would support a life cycle consisting of manufacture–operate–remove–recycle (MORR); however, other options would need to be considered taking into account security, safeguards, market drivers, development tasks, risks and external enablers to create a credible roadmap.

Kidd [33] has observed that there has been a substantial rise in the capital costs of nuclear power plants with a considerable difference between costs in Asia and in Europe and North America leading to many more lower-cost plants being built in China. Some of these differences can be explained by lower labour costs in some parts of Asia; however, the experience gained in building many standardized reactors using financing from state-owned entities is also probably a major factor. Hence, factory production of standardized reactors using automated production techniques offers the opportunity to gain some of these advantages while the lower cost of micro-reactors will reduce the need for and costs of financing.

The scale of the costs of construction of a conventional, large-scale nuclear power plant can be of the order of one-third of the GDP for many countries and if these countries already have gross debts that are a substantial portion of their GDP then their credit rating will impact the costs of financing a nuclear power plant. Perhaps as a consequence, there is an emerging trend towards using private sector finance that has created new challenges due to the uniqueness of each project, the scale of finance required and the timescale of the economic life of the nuclear power plant, all of which require careful consideration in the context the applicability and viability of a proposed finance model [32]. Sainati *et al.* [35] have highlighted the impact of international nuclear law on the ability of private lenders to secure their loans and the bankability of nuclear projects on a non-recourse basis (i.e. repayments are derived only from the profits of the project and not the value of the assets); they concluded that private finance is unlikely to succeed without some form of credit enhancement, such as a credit guarantee scheme of the type used for Hinkley Point C in the UK. Commoditization through a factory-based production and regulatory regime would require substantial investment in the factory which could be borne by a consortium that would realize returns on their investment on a timescale similar to, or shorter than, a single large-scale power station. Substantially fewer factories would be required to support an industrialized economy than large-scale nuclear power stations in the current approach thus reducing the requirement for financing of massive construction projects. The cost of production of individual units would be very much lower than for current nuclear power plants as a result of mass production. Black *et al.* [36] have shown that the base construction cost of a set of 12 light water small modular reactors could be US$_2019_ 2.4 billion for 685 MWe net power output compared to US$_2019_ 6.4 billion for 1147 MWe net power output, i.e. the costs per kilowatt are US$_2019_ 3465 and US$_2019_ 5587, respectively. The SMRs in their case study were not designed for factory production and hence the production costs of the modules and the construction and assembly costs on-site would likely be substantially higher than for type-approved factory-produced micro-power units. Hence, a 20 MWe micro-power unit could have a capital cost of the order of US$ 70 million (≈20 MWe × US$_2019_ 3465 kW^−1^), or less, and be transported to the site, connected to a local grid and generating power, and hence income, in months thus removing many, if not all, of the current financing constraints of nuclear power.

Veenstra *et al.* [37] have highlighted a key economic difference between fossil fuel power plants and nuclear, solar and wind power plants. The former, together with biomass fuelled plants, incur a specific or marginal cost for each unit of electricity generated which is dependent on the market cost of the fuel. Whereas, nuclear, wind and solar power plants incur very small additional costs when producing power; however, the fixed costs from construction, maintenance and decommissioning are a very high proportion of the costs for these types of power plants. These differences render the financial risk for investments in nuclear, solar and wind much higher than for fossil fuel power plants due to both high capital costs and the possibility of insufficient return from selling electricity due to reductions in price and/or demand. The capital cost of factory-produced micro-reactors would be substantially lower thus reducing these risks which would be largely transferred to the investment in the factory. The MORR approach could be deployed through leasing of micro-power units to electricity generators which would provide a stable income stream for the factory and a low threshold to access generating capacity, in a similar manner to that used in the aerospace industry [38,39]. The lower capital costs and immediate availability of micro-power units would make them financially more competitive with wind and solar energy while maintaining the advantage of providing power continuously regardless of weather conditions.

The cost of electricity from different sources is perhaps mostly commonly compared using the levelized cost of energy (LCOE) in which the total cost to build and operate a source of electrical energy is divided by its energy output in kWh [40]. Commoditization is only likely to occur if the LCOE of nuclear power is competitive with other green energy sources, i.e. about 30 US$_2023_ MWh^−1^ for the lowest cost onshore wind (Denmark and Norway) or 35 US$_2023_ MWh^−1^ for utility-scale solar PV in France, India and the USA rather than 70–80 US$_2023_ MWh^−1^ for LWR and Gen III reactors in France, Japan and the USA [41]. Since the construction costs can be as much as 85% of the cost of electricity generation using large-scale nuclear power plants [37], then it is reasonable to assume that the LCOE of nuclear power could be halved by commoditization based on factory-produced micro-power units with substantially lower capital costs due to the shorter timescale from conception to generating electricity.

The ecological cost or footprint of a power plant is also significant and can be assessed using a life cycle assessment (LCA). In 2013, Turconi *et al.* [42] reviewed 167 case studies of LCAs for electricity generation from fossil fuel, hydroelectric, solar and wind as well as nuclear power. They found that while direct emissions from the power plant accounted for the majority of emissions for fossil fuel power plants, for nuclear power the provision of fuel made the largest contribution and for renewable energy sources the creation of the infrastructure contributed the largest proportion of their impact on emissions. Overall, they found that the total life cycle emissions of greenhouse gases, NO_x_ and sulfur dioxide were lower for nuclear power than for all other sources of electricity generation. More recently, a comparative LCA of nuclear, wind and hydropower generation in Ontario, Canada, found that their global warming potential (GWP100) were 3.4, 12.1 and 15.2 g CO_2_-eq kWh^−1^, respectively [43]; while a similar study in China obtained values of 12.4, 28.6 and 3.5 g CO_2_-eq kWh^−1^, respectively [44]. The discrepancies in these data are likely due to differences in technology deployed in the two countries and the definition of the system boundaries. However, in both case studies, the production of the fuel, construction of the nuclear power plant and its decommissioning accounted for most of the global warming potential (in descending contribution) and hence there is an opportunity for an MORR approach to substantially reduce the ecological costs of nuclear power, through lower construction and decommissioning costs, as well as to achieve competitive LCOE values which would likely make a major contribution to gaining stakeholder acceptance.

### 4.2. Socio-political implications

The blueprint for a manufacturing eco-system is very different from traditional one-off nuclear new builds and creates nuclear power as a commodity. As with other commodities, emphasis on assurance and regulation will be centred on the factory with little, if any, demand placed on the sites of distribution or consumers of power. Given this radical shift, communication pathways will need to differ markedly, to maintain stakeholder trust and confidence in the eco-system. Recent work has used social network analysis to identify knowledge management processes and interactions within and between organizations operating and maintaining parts of the current nuclear power industry in the UK [45,46]. This work will need to be extended to identify the knowledge base and social network required to support the MORR concept, which is likely to require a shift towards the type of supply chain that underpins the aerospace industry, another safety-conscious and heavily regulated industry. The civil aerospace industry is a complex ecosystem of manufacturers, suppliers, repairers, maintainers and customers, both airlines and passengers. The ecosystem is diverse with suppliers, consumers and other stakeholders distributed geographically and a correspondingly complex web of digital data. Competition is intense and drives innovation and efficiency from which the nuclear industry is largely protected at the moment but that would be introduced by commoditization. Hence, the importance of identifying suitable social network models, with communication nodes and pathways, for a commoditized civil nuclear power industry.

The appearance of factory-built micro-power units in non-nuclear industrial or urban settings is essentially an act of commoditization. Such micro-power units would need to embody all the features expected of a ubiquitous and unobtrusive presence in our technological landscape: safe and secure; ease of operation; zero end-user maintenance; ruthless uniformity; and a familiar, unchanging ‘look and feel’. Micro-power units delivered within a digital environment under the principles of MORR offer the first genuine prospect of this vision being realized. Digitally enabled factory production not only offers the potential of identically beautiful, ‘user friendly’ micro-power units but also opens up the design and assurance space to much wider contribution and scrutiny of all aspects, including ecological footprint, safety and security. Contemporary strategic infrastructure projects are now recognized as ‘social’ endeavours involving a much broader range of stakeholders than traditionally recognized. The concept of a digital twin/replica as a boundary object between stakeholder and owner/operator would allow the ‘opening up’ of the nuclear energy domain, with a level of transparency entirely unprecedented in such a ruthlessly traditional industry which is frequently misaligned with the communities it seeks to serve, as discussed by Iakovleva [47].

Stakeholder approval of micro-power units as a product will also depend on their integration and acceptance into urban and industrial environments. The use of the ‘ten principles of good design’ embodying the concept of ‘less, but better’, pioneered by the German industrial designer Dieter Rams [48], could be used to create designs for micro-power units that will go unnoticed in an urban environment or even become an iconic product that signifies a community’s commitment to responsible stewardship of the Earth’s resources. Rams believed it was essential that designers work in extremely close cooperation with research and development, engineering production and marketing [49] to create products that reflect a cultural and artistic impulse while drawing attention to themselves by their unobtrusiveness. He proposed this could be achieved through his ten design principles, namely that good design is aesthetic, durable, honest, innovative and unobtrusive as well as making a useful product, helping to understand a product, being consequential to the last detail, concerned with the environment and involving as little design as possible [48].

### 4.3. Technical implications

Alongside the social network described above, a digital network will be required to operate at a number of levels. A reconfigurable high-level architecture (HLA), based on the INDE [17] and NVEC [50] concepts, will be required that is capable of adaptation to changes in product design and/or manufacturing processes throughout the design, manufacture and operational service life of a micro-power unit. The HLA will need (i) to be adaptable to hierarchical changes in coupled or uncoupled design parameters and combinations of process variables; (ii) to provide a framework for the definition and approval of the data and information being passed between subsystems: to include a data and cyber security policy; and (iii) to support visualization of the micro-power unit during its design, certification, manufacture, operation, removal and recycling. As described by Patterson *et al.* [17], the creation of a digital twin of each power unit will enable real-time condition monitoring and control and allow the rapid identification of the cause of any fault in an individual unit so that preventative measures can be quickly implemented across a fleet of units.

The proposed paradigm change from ‘job’ to ‘flow’ production and corresponding regulatory approval of type rather than individual units is radically different from traditional one-off nuclear new builds and converts nuclear power into a commodity. In common with other commodities, the focus of assurance and regulation will be the factory with little, if any, demand placed on the sites of power generation. This implies that communication pathways and processes will need to change in a corresponding manner in order to ensure stakeholder trust and confidence. Digital threads and blockchains could provide these pathways and assurance processes, respectively. Digital threads, also known as digital chains, are the flows of information about the use and performance of a product, in this case of a micro-power unit, throughout its life cycle from design to recycling. Whereas blockchains are decentralized digital databases that store and share information in blocks that are connected together to make up chains. A blockchain is formed by a global network of autonomous computers communicating with one another to reach a consensus on the contents of a database or ledger [51]. The computers compete to be paid to validate the ledger by solving the cryptographic equations that describe a series of transactions. The requirement for the global distribution of autonomous computers means that a blockchain is not lodged at a single location or managed by a single entity or group of entities. This decentralization makes the blockchain both almost incorruptible and expensive in terms of time and energy. Hence, blockchains can be used to authenticate digital information and update it during engineering processes, including manufacture, assembly, operation and recycling, in a way that allows it to be shared with regulators, clients and consumers to form the basis of a quality assurance system.

A key motivation for proposing the commoditization of civil nuclear power is to provide another viable route to sustainable energy production and achieve net zero greenhouse gas emissions by 2050. Hence, it will be important to evaluate and minimize the ecological footprint, i.e. the total demand on natural capital, of the micro-power units functioning in a MORR life cycle. Their ecological footprint could be predicted during the design stage using the digital representation in the HLA described above and subsequently tracked in real life using blockchains to record the contribution of every process during the life cycle.

Waste would need to be a key consideration. While recycling would allow the re-use of a large proportion of the components and structure of the micro-power units thus minimizing lower-level and medium-level nuclear waste, an increase in energy production from nuclear power would inevitably lead to an increase in spent fuel that would need to be processed and stored. This could be achieved at the site of the factory in the short to medium term, as is common practice at conventional large nuclear power plants, prior to long-term storage in geological disposal facilities currently planned or under construction for existing spent fuel in various countries [52,53].

## 5. Discussion

A vision for the commoditization of nuclear power based on factory production of micro-power units has been described with a blueprint for its realization and consideration of the economic, socio-political and technical implications. There are a number of challenges to be considered, including the design of both a factory and its supply chain; the creation of trust among a supply chain, regulatory system, owners, operators and the general public; the establishment of a new regulatory regime based on approving the design, production and quality assurance processes for a type or series of micro-power units; and the relationship with the general public, who would need to perceive nuclear power as a desirable asset with acceptable financial and ecological costs. These are discussed in the following sections.

### 5.1. Cultural changes

A recent systematic literature review of public opinion related to energy infrastructure found that individuals are more often supportive of energy projects than they are opposed with knowledge, trust and positive perception about the benefits of projects being correlated positively with support [54]. Public opinion relating to civil nuclear power varies by country and with time, as shown recently by Yamagata in a case study focused on Japan, the UK and the USA [55]. For many decades in the UK, the provision of new nuclear reactors has been couched in the language of ‘acceptance’ through education. Commoditization allows for a much more ambitious approach to nuclear energy as a social practice. The opening up of digital interaction spaces will create new, emergent publics which will fundamentally change the debate around nuclear provision. Although not all the implications are entirely predictable, this radical increase in inclusivity is certain to introduce more diverse voices and expertise into the debate and close, or in some cases eliminate, the gap between providers and consumers. Research is required to explore the most appropriate and effective approach to utilizing digital technologies to engage and bring together all stakeholders, as has been achieved, for example, in Helsinki [56] and Herrenberg [57] using digital representations of each city as a platform for co-design and development with its citizens.

Increasingly, new nuclear projects are being packaged as ‘partnerships’ between development companies and host communities [58,59]. This arrangement should imply not only an increased influence over any given project by a local community but also, by implication, an increased accountability for its success. Such accountability would be expected to move a host community away from a position of passive acceptance into a much more active and even proactive role as an advocate of nuclear energy. This potential shift has fundamental implications for the nature of communication within such projects. Traditional nuclear energy projects have been characterized by communication pathways that are dissemination-based and unidirectional towards the host community whereas recent work has shown that public participation in communication about nuclear power projects positively affects public acceptance [60,61]. The twin factors of increased community accountability and greater use of digital tools will remap social networks in a way that will likely supplement and/or reformulate formal hierarchical structures. The technique of social network analysis has been used to map the social networks that exist within parts of the electricity generation industry and its supply chain [46]; however, further work is needed to enhance our understanding of these networks and facilitate their transformation to support the commoditization of nuclear power.

There will be another inevitable controversy around the commoditization of nuclear power provision. Commoditization introduces end-user-focused commercial drivers alongside those of energy security and the overriding imperative of achieving net zero. Adding in wider public concerns around the safety and security of nuclear energy highlights the necessity for a different way to navigate the ethical landscape around future commoditization. A new framework of ‘Energy Justice’ has been proposed as a way to underpin the righteousness of energy provision [62]. This framework allows an assessment of any proposal to be made against the general principles of distribution, participation and recognition and has been used to successfully underpin new build schemes across the world [63–65]. The tenets of Energy Justice require a high level of transparency to stakeholders such that benefits and risks can be illustrated and understood. The digitally enabled approach outlined here would provide such a platform and a digital twin of any micro-power unit could form a live and evolving demonstrator of justice throughout the project life cycle.

A paradigm change in the regulatory regime for nuclear power is implied by commoditization which requires a shift to approval of a type or series of micro-power units based on the design principle of containing the consequences of all hazards on-site or ‘in the box’. This type of approach to regulatory approval already exists, for example in the aerospace industry, and hence needs to be translated rather than invented. However, it would require the creation of new regulatory guidelines for civil nuclear power plants and also for the factories and supply chains producing the micro-power units. The scale of change from the present regulatory regime is massive and will need the support of national and international governance organizations. It will have wide-ranging implications that need to be carefully considered together with the potential interactions between the complex technical and social systems. The advent of industrial metaverses provides powerful tools for examining these issues safely in a virtual environment and in relatively short timescales [66,67]. However, research is required to develop these new tools and adapt them to the design, development and testing of a new regulatory regime. It seems likely that in most countries this paradigm shift could not be undertaken utilizing the existing regulatory framework and instead a new one will have to be implemented.

### 5.2. Technical challenges

Most of the technology required to design micro-power units within the US DoE classification of Hazard Category 2 exists and a number of designs are being developed, e.g. Mitsubishi [15]. Further work is required to build and test prototypes. This is relatively straightforward compared to the research and development required to design and build a factory for the flow production of micro-reactors which would be the first-of-a-kind with all of the associated challenges, including the validation of computational models used in the design process without data from real-world full-scale prototypes [18]. Hence, a major programme of research and development is required to develop concept designs, select the best concepts and develop them further prior to selecting and embodying the best design and starting construction. A timeline and level of investment comparable to current investments in commercial fusion energy plants is probably necessary. In addition, the rapid development of digital tools, such as blockchains, is required to underpin the quality assurance processes required for type approval within a radically different regulatory regime. Blockchains would allow digital information to be authenticated and updated during engineering processes, including manufacture, assembly, operation and recycling, so that regulators, clients and consumers can access and review it thus providing trust among all stakeholders in the quality assurance framework.

Levers *et al.* [68] have proposed that digital twins should be considered digital products in their own right. In this context, each micro-power unit would have its physical manifestation produced in a factory and simultaneously a cyber twin, with dynamic connectivity to its physical counterpart, built in an integrated digital environment containing digital representations of the manufacturing processes. Data from the quality assurance processes performed during the production of the physical unit would be provided to its cyber twin. Subsequently, condition monitoring data and operational information would be continuously supplied to the cyber twin so that it remained a reliable digital representation of its physical counterpart. This would allow it to be used for training operators, scenario planning, maintenance planning, life cycle prognosis and exploring interactions with other complex engineering infrastructures via their digital representations, ultimately in an industrial metaverse [69]. Digital twins of each micro-power unit would become individual to each unit and provide a digital archive of its production, maintenance and service life as well as being a simulation tool providing feedback on plant performance and enhancing confidence in its safe operation.

## 6. Conclusions

The commoditization of fission-based nuclear power has been proposed and is based on the factory production of sealed, contained micro-power units whose hazards are contained on-site. This paradigm shift would require a corresponding transformation of the regulatory regime to an approach based on type or series approval that could be supported by a digitally enabled holistic quality assurance framework. This framework would support the safe and secure construction, operation, maintenance and decommissioning of the micro-power units. The deployment has been described of recently developed digital tools, such as industrial metaverses, to support design, development and demonstration of integrated technical and social systems, as well as blockchains to support the quality assurance processes within a supply chain and underpin type approval. The economic, socio-political and technical implications of the commoditization of nuclear power have been discussed. It is likely that factory production of micro-power units, as part of an MORR sequence, would shift the financial risks from the power plant to the factory thereby making nuclear power a realistic competitor for solar and wind power in terms of both financial and ecological costs. The potential barriers, particularly in terms of investment in research and development, are identified and some solutions are offered. The analysis makes it apparent that, while there are some significant technical challenges in moving from ‘job’ production on-site to ‘flow’ production in a factory, the major obstacles to the commoditization of nuclear power are related to the prevailing industrial culture. The proposed production rate and safety regime represent a paradigm change for the nuclear industry that will require a change in design philosophy, development of a digitally assured supply chain and production systems, which in turn will lead to the commoditization of nuclear power.

## Data Availability

This article has no additional data.
